# Knowledge of cervical cancer and HPV vaccine in Bangladeshi women: a population based, cross-sectional study

**DOI:** 10.1186/s12905-018-0510-7

**Published:** 2018-01-11

**Authors:** Jessica Yasmine Islam, Fatema Khatun, Anadil Alam, Farhana Sultana, Afsana Bhuiyan, Nazmul Alam, Laura Reichenbach, Lena Marions, Mustafizur Rahman, Quamrun Nahar

**Affiliations:** 1 Department of Epidemiology, Gillings School of Global Public Health, University of North Carolina at Chapel Hill, Chapel Hill, North Carolina, USA; 2 International Centre for Diarrhoeal Disease Research, Bangladesh (icddr,b), 68 Shaheed Tajuddin Ahmed Sharani, Mohakhali, Dhaka, 1212 Bangladesh; 3 Centre for Epidemiology and Biostatistics, Melbourne School of Population and Global Health, The University of Melbourne, 207 Bouverie Street, Parkville, VIC 3010 Australia; 4 Registries and Research, Victorian Cytology Service Registries, Level 6, 176 Wellington Parade, East Melbourne, VIC 3002 Australia; 5 London North West Healthcare NHS Trust, London, UK; 6 University of Montreal Hospital Research Centre (CR-CHUM), Montreal, QC, Canada; 7 Population Council, Washington, DC USA; 8 Department of Clinical Science and Education, Karolinska Institutet, Stockholm, Sweden

**Keywords:** Knowledge, Cervical cancer, Bangladesh, HPV vaccine

## Abstract

**Background:**

The objective of this study was to assess the level of knowledge of cervical cancer among Bangladeshi women and to assess their willingness to receive the human papillomavirus (HPV) vaccine.

**Methods:**

A population-based, cross-sectional survey was conducted from July to December 2011 in one urban and one rural area of Bangladesh. A total of 2037 ever-married women, aged 14 to 64 years, were interviewed using a structured questionnaire. Data on socio-demographic characteristics and knowledge of cervical cancer were collected. Willingness to receive the HPV vaccine was assessed. Univariate analyses were completed using quantitative data collected. Multivariable logistic regression models were developed to identify factors associated with having heard of cervical cancer and the HPV vaccine.

**Results:**

The majority of study participants reported to have heard of cervical cancer (urban: 89.7%, rural 93.4%; *P* = 0.003). The odds of having heard of cervical cancer were significantly higher in urban women aged 35–44 years (aOR: 2.92 (1.34–6.33) and rural women aged 25–34 years (aOR: 2.90 (1.24–6.73) compared to those aged less than 24 years. Very few women reported to have detailed knowledge on risk factors (urban:9.1%, rural: 8.8%) and prevention (urban: 6.4%, rural: 4.4%) of cervical cancer. In our sample, one in five urban women and one in twenty rural women heard about a vaccine that can prevent cervical cancer. Among urban women, secondary education or higher (aOR: 3.48, 95% CI: 1.67–7.25), age of 20 years and above at marriage (aOR: 2.83, 95% CI: 1.61–5.00), and high socioeconomic status (aOR: 2.25, 95% CI: 1.28–3.95) were factors associated with having heard of the HPV vaccine. Willingness to receive the HPV vaccine among study participants either for themselves (urban: 93.9%, rural: 99.4%) or for their daughters (urban: 91.8%, rural: 99.2%) was high.

**Conclusions:**

Detailed knowledge of cervical cancer among Bangladeshi women was found to be poor. Education on cervical cancer must include information on symptoms, risk factors, and preventive methods. Despite poor knowledge, the study population was willing to receive the HPV vaccine.

## Background

Globally, cervical cancer is the fourth most common cancer among women, with an estimated 528,000 new cases in 2012 [1]. A large majority of the global burden of cervical cancer occurs in less developed regions, where almost nine in ten (87%) deaths are due to cervical cancer [1]. Such disproportionately high mortality rates have been attributed to several factors, including lack of availability of organized and high quality cervical cancer screening and treatment programs, lack of knowledge of cervical cancer, attitudinal barriers, and poor health systems infrastructure [2–5]. In Bangladesh, cervical cancer is the second most common cancer amongst women with an estimated 11,956 incident cases and 6582 deaths in 2012 [6].

Human papillomavirus (HPV) is the necessary cause of cervical cancer [7]. Persistent infection of approximately 15 high-risk HPV genotypes cause almost all cases of cervical cancer and its immediate precursor lesions [8]. Of these genotypes, HPV-16 and HPV-18 account for about 70% of global cervical cancer cases, with HPV-16 causing about 55–60% and HPV-18 about 10–15% [9]. Similarly, according to our previously published findings, HPV-16 is the most common high-risk HPV genotype detected in Bangladeshi women [10]. This consistent global discovery has resulted in the major development of primary prevention of cervical cancer through HPV vaccination of young adolescents [11].

Currently, there are three types of HPV vaccines available in the global market: nonavalent vaccine “Gardasil – 9™,” quadrivalent “Gardasil™” and bivalent “Cervarix™.” The HPV vaccine has demonstrated high degrees of efficacy with maximum clinical effectiveness and cost-effectiveness in the target population of young adolescents, who are less likely to have been previously exposed to high-risk HPV genotypes [12, 13]. As these vaccines are highly effective before exposure to HPV, current guidelines prioritize adolescent girls as the primary target group for HPV vaccination [14].

While the vaccine has been shown to be efficacious, high vaccine uptake is essential for successful HPV vaccine program implementation [15]. Several factors have been attributed to high vaccine uptake among women, including knowledge of cervical cancer and the HPV vaccine, and its associated benefits [15], specifically in the United States (U.S.) and Australia. Additional studies conducted in the U.S. have shown that caregivers of vaccinated children are more likely to be knowledgeable of the HPV vaccine than caregivers of non-vaccinated children [16, 17]. However, research conducted in developing countries, including Kenya and Nepal, have shown high HPV vaccine acceptability and willingness to receive the vaccine among women, despite low to moderate levels of knowledge of cervical cancer and the HPV vaccine [18, 19]. Country-level data on knowledge of cervical cancer and attitudes towards the HPV vaccine among Bangladeshi women are imperative for resource allocation of cervical cancer primary prevention programs and to identify target groups for future educational programs. Data are particularly necessary as the HPV vaccine has been recently introduced in 2016 for the first time in Bangladesh by the Ministry of Health, with support from the Global Alliance for Vaccines and Immunizations (GAVI) [20].

Among Bangladeshi women, one previous report has documented high (81%) awareness of cervical cancer, however, the majority (74%) of these data were collected from women residing in rural areas and limited to women above the age of 30 years [21]. Additionally, in-depth knowledge on cervical cancer, such as risk factors or symptoms, was not assessed. Data on knowledge of cervical cancer among a more representative sample, including those residing in urban areas and younger populations, are needed. Additionally, data on knowledge of HPV and the HPV vaccine among Bangladeshi women are currently not available in the literature. Therefore, the objective of this study was to assess women’s knowledge of cervical cancer and willingness to receive the HPV vaccine among women residing in both rural and urban areas in Bangladesh. Additionally, factors associated with having heard of cervical cancer and the HPV vaccine were identified by urban and rural area of residence among this population. We hypothesized that knowledge of cervical cancer will be low and willingness to receive the HPV vaccine will be high, similar to other developing countries in the region.

## Methods

### Study population

Data for this study were collected during the baseline assessment of a population-based longitudinal cohort study developed to estimate the overall burden of HPV infection and risk factors associated with persistent infection among females in Bangladesh. This cross-sectional survey was conducted between July and December of 2011 in one rural and urban area of Bangladesh. The urban area was located in Dhaka, the capital of Bangladesh. Since 2010, the International Centre for Diarrhoeal Disease Research, Bangladesh (icddr,b), an international research organization, has maintained a surveillance site for on-going research projects in Dhaka in one specific administrative ward (smallest administrative urban geographic unit), which covers a population of approximately 27,000. To construct the sampling frame for the urban site, the administrative ward was divided into nine clusters, of which three were selected for this study. The rural area was located in Mirzapur, a sub-district of Tangail district. In Tangail, icddr,b has organized a demographic surveillance site since 2007. This surveillance site includes a population of approximately 240,000 and consists of eight unions (smallest administrative rural geographic unit). Of these eight unions, two were purposely selected and included in the sampling frame for the rural site. Systematic random sampling technique was used to select women in both areas.

Women who were ever-married, aged 14–64 years and residing in the study areas were invited to the study clinic for further assessment by the study physician. Women who were pregnant, post-partum, or with a history of hysterectomy, cervical cancer, uterine prolapse or with major psychological/psychiatric problems were excluded after history taking and/or clinical examination by the study physician. Pregnant women and women who were post-partum were excluded as cervical sampling implements may cause bleeding and interfere with the HPV test results. Further details regarding sampling methods, data collection, HPV testing and survey implementation methods have been previously published [10].

### Survey instrument

Upon providing consent, women were interviewed using a structured and close-ended questionnaire, which collected demographic information, reproductive history, lifestyle factors such as condom and tobacco use, and knowledge of cervical cancer and attitudes towards the HPV vaccine. In order to assess knowledge of cervical cancer, participants were asked if they had “ever heard of a cancer called cervical cancer?” If participants responded, “Yes,” then a series of questions to assess women’s knowledge related to the disease and attitudes towards the HPV vaccination were asked, however, if a participant responded “No” then knowledge of cervical cancer and attitudes towards the HPV vaccine was not further assessed.

In order to assess knowledge, the following questions were asked: (1) “From where did you hear about cervical cancer?”; (2) “Do you know how a woman can get this cancer?”; (3) “If yes, how can a woman get this cancer?”; (4) “Do you think cervical cancer can be prevented?”; and (5) “If yes, how can it be prevented?” Possible responses to all knowledge questions were defined in the questionnaire, including an “Other” option. Participants were able to provide a free response if they chose “Other.” Responses to “Other” were recoded and included in the analysis. Participants were able to provide more than one response for each question.

Next, participants were asked a series of questions to assess their attitudes towards the HPV vaccination. If a participant had previously indicated they had never heard of cervical cancer, then these questions were skipped. Questions related to attitudes towards the HPV vaccination included, (1) “Have you heard of a vaccine to prevent cervical cancer?”; (2) “Would you support a measure to prevent cervical cancer?”; (3) “If there was such a vaccine available, would you take the vaccine to prevent cervical cancer?”; (4) “Would you be willing to give your daughter such a vaccine?”; and (5) “Would you be willing to recommend the vaccine to others?” Participants were able to answers these questions with either “Yes,” “No” or “Not Sure/Don’t Know.”

### Sample size

The sample size necessary to carry out this study was calculated using the parent study’s objective to assess the prevalence of HPV in the Bangladeshi population. Prior to our study, the prevalence of HPV in Bangladesh was unknown. Based on estimates of HPV prevalence from neighboring countries, we assumed the prevalence of HPV infection among the general female population in Bangladesh would be close to 8%. We utilized the following formula to calculate the sample size [22]:

$$ n=\frac{Z^2P\ \left(1-P\right)}{d^2} $$

Here n = sample size, Z = Z statistic for a level of confidence, P = expected prevalence or proportion and d = precision. Using this formula, we calculated the necessary sample size for the urban and rural sites separately to account for potential differences in loss to follow-up. For both sites we used the following parameters to complete our estimates: 8% prevalence, 2% allowable error, and 95% confidence intervals. For the rural site, we estimated our loss to follow-up may reach 20%, including drop out due to pregnancy during the two-year follow-up period leading to a target sample size of 883. For the urban site, we assumed loss to follow-up may reach 30%, as residents of urban areas are more mobile than rural areas. Based on this assumption, the target sample size for the urban site was 1009 women.

### Data analysis

Descriptive analyses were conducted to assess demographic characteristics of participants, by rural and urban area of residence. Frequency distributions were presented on background characteristics, including age, marital status, education, occupation, parity, and monthly household expenditure as a proxy measure for socioeconomic status. Test of significance was performed using Chi-square test at 5% level of significance. Multivariable logistic regression models were generated separately for urban and rural women to assess factors associated with “Ever heard of Cervical cancer” and “Ever heard of HPV vaccine” as dependent variables. Adjusted odds ratios (aORs) and its 95% confidence intervals (CIs) were estimated. Variables of interest to include in the multivariable model were initially identified using the existing literature [23, 24]. Next, these variables were assessed using univariate analyses. Any factor that provided a univariate *p*-value <0.05 for either the rural or urban model was included in the final multivariable regression models. The following variables were adjusted for in the models: age, education, occupation, age at marriage, husband’s education, parity, socioeconomic status, oral pill use, abortion history, and condom use. Collinearity was assessed using the variance inflation factor (VIF) to ensure a strong linear relationship among independent variables included in the model was not present. Due to the sampling design and possible effect modification, data analyses were carried out separately for urban and rural samples. All statistical analyses were performed using STATA/SE 12.0.

## Results

A total of 3354 eligible women were approached; 1855 in the urban area and 1499 in the rural area. Of the 1855 urban women approached, 1152 (62%) agreed to participate. Among those who agreed to participate, 1113 (97%) completed interviews. In the rural areas, 1159 of 1499 (77%) eligible women agreed to participate and 924 (80%) completed interviews. Common reasons included refused to take part in the study (*n* = 597; 18%), failure to attend scheduled interview located at project office (*n* = 330; 10%) or were found ineligible based on study exclusion criteria (pregnant, post-partum, or with a history of hysterectomy, cervical cancer, uterine prolapse or with major psychological/psychiatric) after examination conducted by the study physician at enrollment (*n* = 390; 12%).

### Background characteristics

Table 1 presents socio-demographic characteristics of our study population in urban and rural areas in this study. Urban and rural women differed significantly on most socio-demographic characters except parity. In both urban and rural sites, the average age of women was 33 years. Significantly more women in the rural area reported they were married than their urban counterpart (98% versus 90%; *p* < 0.001). Overall, women from the urban site were more educated, with an average of approximately 7 years of education compared to 5 years of education among rural women. 16% of urban women reported to work as a garment worker or housemaid compared to about 1% of rural women. Additionally, a higher proportion of urban women (31%) reported a monthly expenditure of 20,000 BDT or greater compared to rural women (7%). One-third of rural women were married at 14 years of age or younger. Urban women reported to have more educated husbands than rural women with an average of approximately 9 years’ education compared to about 6 years respectively (*p* < 0.001). In our sample, significantly more women in rural areas heard of cervical cancer compared to urban women (93% versus 90%, *p* = 0.003). Very few women reported to have ever had a cervical cancer screening test. There were significantly more women in the urban area who underwent cervical cancer screening compared to their rural counterpart (3% versus 1%, p < 0.001). None of the women in this study reported to have previously received the HPV vaccine, based on self-report.

**Table 1:** Demographic characteristics of study population to assess knowledge of cervical cancer and the Human Papillomavirus (HPV) vaccine among ever-married Bangladeshi women (*n* = 2037)

	Urban (*n* = 1113)		Rural (*n* = 924)		
Characteristics	*n*	%	*n*	%	*P*-Value^a^
Age (in years)					0.001
ᅟ< 24	242	21.7	195	21.2	
ᅟ25–34	432	38.8	328	35.5	
ᅟ35–44	246	22.1	275	29.8	
ᅟ45–64	193	17.3	126	13.6	
Mean Age ± SD	33.1 ± 10.3		33.1 ± 8.9		
Marital Status					<0.001
ᅟMarried	997	89.6	905	97.9	
ᅟSeparated/Divorced/ Widowed	116	10.4	19	2.1	
Religion					<0.001
ᅟMuslim	1098	98.7	796	86.1	
ᅟNon-Muslim	15	1.4	128	13.6	
Education					<0.001
ᅟNo education	212	19.1	272	29.4	
ᅟPrimary	270	24.3	244	26.4	
ᅟSecondary or more	631	56.7	408	44.2	
Mean Education ± SD	6.7 ± 4.6		4.9 ± 3.9		
Occupation					<0.001
ᅟHousewife	769	69.1	860	93.1	
ᅟGarments worker / Housemaid	176	15.8	6	0.7	
ᅟOther work^b^	168	15.1	58	6.3	
Socioeconomic Status^c^					<0.001
ᅟLow (<10,000)	248	22.3	542	58.7	
ᅟMedium (10,000–19,999)	526	47.3	320	34.6	
ᅟHigh (20,000+)	339	30.5	62	6.7	
Age at Marriage (in years)					<0.001
ᅟ < = 14	282	25.3	313	33.9	
ᅟ15–19	579	52	515	55.7	
ᅟ20 or more	252	22.6	96	10.4	
Mean Age at Marriage ± SD	17.1 ± 3.4		16.0 ± 2.8		
Husband’s Education					<0.001
ᅟNo Education	125	11.2	294	31.8	
ᅟPrimary	157	14.1	206	22.3	
ᅟSecondary or more	831	74.7	424	45.9	
Mean Education ± SD	8.9 ± 4.8		5.5 ± 4.5		
Parity					0.612
ᅟ0	76	6.8	63	6.8	
ᅟ1–2	451	40.5	394	42.6	
ᅟ3 +	586	52.7	467	50.5	
Mean Parity ± SD	2.9 ± 2.1		2.7 ± 1.7		
Oral Pill Usage					
ᅟEver	874	78.5	799	86.5	<0.001
ᅟNever	239	21.5	125	13.5	
Abortion History					
ᅟ Ever	417	37.5	91	9.9	<0.001
ᅟ Never	696	62.5	833	90.2	
Tobacco Use					
ᅟ Ever	292	73.8	235	74.5	0.681
ᅟ Never	821	26.2	689	25.4	
Condom Use					
ᅟEver	574	51.6	301	32.6	<0.001
ᅟNever	539	48.4	623	67.4	
Ever Heard of Cervical Cancer					0.003
ᅟYes	998	89.7	863	93.4	
ᅟNo	115	10.3	61	6.6	
Ever Underwent Cervical Cancer Screening					<0.001
ᅟYes	34	3.1	6	0.7	
ᅟNo	1079	96.9	918	99.4	

^a^Calculated using chi-squared test

^b^Includes government and private service, business, daily wager, agriculture, tailor, poultry, handicrafts and tutor

^c^Monthly Expenditure BDT, 1US$ = 78 BDT

### Knowledge of cervical cancer and willingness to receive HPV vaccine

Of the women who reported to have heard of cervical cancer (*n* = 1861), only one in ten reported knowing different ways of getting the disease (*n* = 182) (Table 2). Urban women reported the most common way of developing cervical cancer was sexual intercourse (33%), which in rural women was “poor hygiene during menstruation” (22%). “Sexual relationship other than husband” was the next common way of getting cervical cancer in both rural (20%) and urban (21%) women. Very few women (urban: 6%, rural: 3%) indicated, “not using a condom” as a way to develop cervical cancer.

**Table 2:** Knowledge of ever-married Bangladeshi women of main risk factors and prevention measures associated with cervical cancer (*n* = 1861) ^a^

	Urban		Rural	
	n	%	n	%
Ways to Develop Cervical Cancer (*n* = 182) ^b^				
Sexual Intercourse	33	32.7	11	13.6
Sexual relationship with other than husband	21	20.8	16	19.8
Poor hygiene during Menstruation	18	17.8	18	22.2
General poor hygiene	6	6.0	8	10.0
Husband has a disease	5	4.9	4	4.9
Food / Water	1	0.9	3	3.7
Not using a condom	6	5.9	2	2.5
Skin-to-skin contact	3	2.9	9	11.1
Preventive Methods against Cervical Cancer (*n* = 121) ^c^				
Sexual relationship only with husband	23	32.4	8	19.5
Cleanliness	22	30.9	18	43.9
Vaccination	11	15.5	2	4.9
One-time examination by Doctor	10	14.1	5	12.2
Condom Use	9	12.7	3	7.3
Regular Examination by doctor	3	4.2	0	0.0
Medications	2	2.8	13	31.7
Eating Well	1	1.4	0	0.0
Source of Knowledge of Cervical Cancer				
Neighbors	549	55.0	572	66.3
Relatives (besides husband)	304	30.5	389	45.1
Television	301	30.2	127	14.7
Doctor	123	12.3	66	7.7
Other Health Professional	126	12.6	66	7.7
News paper	45	4.5	4	0.5
Colleagues	29	2.9	5	0.6
Radio	12	1.2	5	0.6
Friends	13	1.3	1	0.1
Husband	3	0.3	8	0.9

^a^Percent may exceed 100% as multiple answers are possible and sample is limited to those who reported to have heard of cervical cancer

^b^Sample is limited to those who reported to know how a woman can get cervical cancer (*n* = 182)

^c^Sample is limited to those who reported to know about preventive methods for cervical cancer (*n* = 121)

Women’s knowledge about prevention of cervical cancer was also very poor. Very few women believed that cervical cancer can be prevented (urban: 7%, rural: 4%). When asked about the ways this disease can be prevented, the majority thought that cleanliness (urban: 31%, rural: 44%) and maintaining a monogamous sexual relationship with their husband (urban: 32%, rural: 20%) was the most effective preventive measure. Over one-third of rural women mentioned medications as a method to prevent cervical cancer. 16% of urban women mentioned vaccination as a preventive method against cervical cancer compared to 5% of rural women. Very few women, both in urban and rural areas, were able to indicate “regular examination by doctor” as a method to prevent cervical cancer (Table 2). Of the 1861 women (91%) who reported to have heard of cervical cancer, the majority of women heard about it from neighbors (urban: 55%, rural: 66%), relatives (urban: 31%, rural: 45%) and television (urban: 30%, rural: 15%) (Table 2).

Table 3 presents results from the multivariable logistic regression models of factors associated with having heard of cervical cancer. After controlling the effects of other factors in the models, we identified an effect of age among both urban and rural samples. Among urban women, the odds of having heard of cervical cancer were highest among those aged 35–44 years when compared to those aged less than 24 years (aOR: 2.92, CI: 1.34–6.33, *p* = 0.007). In rural women, the odds of having heard of cervical cancer were significant only for the age group 25–34 years when compared to those aged less than 24 years (aOR: 2.90, CI: 1.24–6.73, *p* = 0.014).

**Table 3:** Factors associated with having heard of cervical cancer among ever-married Bangladeshi women (*n* = 2037)

	Urban Population (*n* = 1113)				Rural Population (*n* = 918)			
	Crude OR^a^	*P*	Adjusted OR^b^	*P*	Crude OR^a^	*P*	Adjusted OR^b^	*P*
Age (in years)								
ᅟ < 24	Ref.		Ref.				Ref.	
ᅟ25–34	4.09 (2.52–6.61)	<0.001	2.73 (1.54–4.82)	0.001	2.84 (1.34–5.99)	0.006	2.90 (1.24–6.73)	0.014
ᅟ35–44	4.23 (2.35–7.62)	<0.001	2.92 (1.34–6.33)	0.007	1.45 (0.75–2.82)	0.269	1.87 (0.72–4.84)	0.198
ᅟ45–64	3.49 (1.90–6.40)	<0.001	2.55 (1.06–6.12)	0.036	1.13 (0.52–2.46)	0.761	1.72 (0.56–5.32)	0.345
Education								
ᅟNo education	Ref.		Ref.		Ref.		Ref.	
ᅟPrimary	3.12 (1.77–5.52)	<0.001	2.44 (1.33–4.49)	0.004	1.88 (0.93–3.80)	0.079	1.59 (0.75–3.35)	0.228
ᅟSecondary or more	6.57 (3.81–11.33)	<0.001	3.76 (1.97–7.18)	<0.001	3.52 (1.66–7.45)	0.001	2.18 (0.91–5.22)	0.080
Occupation								
ᅟHousewife	Ref.		Ref.		Ref.		Ref.	
ᅟGarments worker / Housemaid	0.38 (0.24–0.60)	<0.001	0.86 (0.52–1.45)	0.588	–		–	ns
ᅟOther work^c^	0.92 (0.49–1.74)	0.804	0.92 (0.47–1.80)	0.814	3.59 (0.48–26.62)	0.211	3.02 (0.40–22.97)	0.286
Age at Marriage (in years)								
ᅟ ≤ 14	Ref.		Ref.		Ref.		Ref.	
ᅟ15–19	0.94 (0.58–1.54)	0.818	0.78 (0.46–1.34)	0.514	1.24 (0.71–2.16)	0.446	0.89 (0.49–1.61)	0.701
ᅟ ≥ 20	1.96 (0.97–3.97)	0.06	1.40 (0.65–3.05)	0.388	2.42 (0.71–8.27)	0.158	1.58 (0.45–5.63)	0.477
Husband’s Education								
ᅟNo Education	Ref.		Ref.		Ref.		Ref.	
ᅟPrimary	1.36 (0.72–2.58)	0.349	0.98 (0.49–1.94)	0.954	1.26 (0.66–2.42)	0.488	1.03 (0.52–2.05)	0.927
ᅟSecondary or more	3.88 (2.22–6.77)	<0.001	1.67 (0.88 - 3.19)	0.115	2.96 (1.52–5.75)	0.001	1.89 (0.88–4.08)	0.104
Parity								
ᅟ0	Ref.		Ref.		Ref.		Ref.	
ᅟ1–2	1.48 (0.80–2.73)	0.210	1.98 (0.99–3.94)	0.051	1.17 (0.45–3.05)	0.747	1.22 (0.46–3.28)	0.680
ᅟ3 +	2.68 (1.27–5.64)	0.01	5.28 (2.12–13.15)	<0.001	1.48 (0.48–4.55)	0.496	1.70 (0.53–5.47)	0.370
Socioeconomic Status^d^								
ᅟLow (<10,000)	Ref.		Ref.		Ref.		Ref.	
ᅟMedium (10,000–19,999)	2.31 (1.50–3.57)	<0.001	1.51 (0.94–2.42)	0.086	1.49 (0.83–2.67)	0.183	1.20 (0.66–2.20)	0.541
ᅟHigh (20,000+)	6.43 (3.22–12.82)	<0.001	3.00 (1.42–6.34)	0.004	2.62 (0.62–11.15)	0.192	1.28 (0.28–5.78)	0.748
Oral Pill Usage								
ᅟNever	Ref.		Ref.		Ref.		Ref.	
ᅟEver	0.93 (0.57–1.52)	<0.001	0.89 (0.46–1.37)	0.408	1.81 (0.93–3.52)	0.082	1.40 (0.68–2.84)	0.359
Abortion History								
ᅟNever	Ref.		Ref.		Ref.		Ref.	
ᅟEver	0.88 (0.58–1.34)	0.552	0.61 (0.38–1.00)	0.051	2.20 (0.66–7.32)	0.197	1.44 (0.41–4.97)	0.567
Condom Usage								
ᅟNever	Ref.		Ref.				Ref.	
ᅟEver	2.42 (1.55–3.76)	<0.001	1.66 (1.03–2.67)	0.036	2.17 (1.12–4.19)	0.021	1.53 (0.77–3.05)	0.229

^a^Adjusted for age

^b^Adjusted for age, education, occupation, age at marriage, parity, oral pill usage, abortion history, socioeconomic status (income), condom use, husband’s education

^c^Includes government and private service, business, daily wager, agriculture, tailor, poultry, handicrafts and tutor

^d^Monthly Expenditure BDT, 1US$ = 78 BDT

Education was identified as a significant factor among urban women. Urban women with primary education (aOR: 2.44, CI: 1.33–4.49, *p* = 0.004) and secondary education or above (aOR: 3.76, CI: 1.97–7.18, *p* < 0.001) had increased odds of hearing of cervical cancer when compared to urban women with no education. No significant effect of education was observed among rural women.

Parity was found to be associated with having heard of cervical cancer for urban women only. In particular, the odds of having heard of cervical cancer among urban women with three children or more was 5 times that of urban women with no children (aOR: 5.28, CI: 2.12–13.15, *p* = <0.001). Similarly, socioeconomic status was only associated with the having heard of cervical cancer among urban women. The odds of the outcome among urban women with a high socioeconomic status (SES) was 3.0 times that of the odds of urban women with a low SES (aOR: 3.00, CI: 1.42–6.34, *p* = 0.004). And finally, ever condom usage among urban women was a significant factor associated with having heard of cervical cancer; the odds of having heard of cervical cancer was 67% higher among urban women who reported to have ever used a condom when compared to those who did not.

### Attitude towards HPV vaccination

**Fig. 1:**
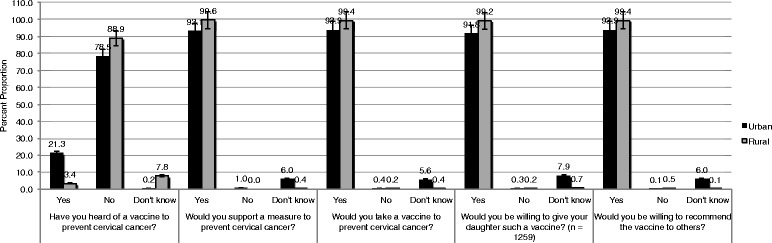
Willingness to vaccinate against cervical cancer among ever-married Bangladesh women (*n* = 1861)

Twenty-one percent of urban women and only 3% of rural women reported to have heard of a vaccine that can prevent cervical cancer (Fig. 1). When asked if they would take a vaccine to prevent cervical cancer, almost all women said yes (urban: 94%, rural: 99%). The majority of women were also willing to recommend the vaccine to others (urban: 92%, rural: 99%). Participants who had a daughter were asked if they would be willing to provide such a vaccine to their daughters to which most women said yes (urban: 92%, rural: 99%) (Fig. 1).

Table 4 presents results of the multivariable logistic regression models to identify factors associated with having heard of the HPV vaccine. Overall, no association was identified with age after adjusting for covariates previously identified to be associated with the outcome among both urban and rural women. Education was associated with the outcome among urban women only; the odds of having heard of HPV vaccine among urban women with secondary education or above was 3.48 times the odds of those with no formal education (aOR: 3.48, 95% CI: 1.67–7.25, *p* = 0.001). Similarly, age at marriage was only associated with the outcome among urban women only. Among those married at both 15–19 years (aOR: 2.18, 95% CI: 1.32–3.60, *p* = 0.002) and 20 years or above (aOR: 2.83, 95% CI: 1.61–5.00, *p* < 0.001), the odds were more than doubled when compared to those married at 14 years or below. Finally, high socioeconomic status was associated with having heard of HPV vaccine among urban women as well (aOR: 2.25, 95% CI: 1.28–3.95, *p* = 0.005).

**Table 4:** Factors associated with having heard of HPV Vaccine among ever-married Bangladeshi women (*n* = 2037)

	Urban Population (*n* = 1113)				Rural Population (*n* = 918)			
	Crude OR^a^	*P*	Adjusted OR^b^	*P*	Crude OR^a^	*P*	Adjusted OR^b^	*P*
Age (in years)								
< 24	Ref.		Ref.		Ref.		Ref.	
25–34	1.54 (1.01–2.36)	0.042	1.13 (0.68–1.89)	0.632	1.57 (0.55–4.47)	0.4	1.30 (0.39–4.36)	0.674
35–44	1.25 (0.77–2.01)	0.37	0.93 (0.50–1.73)	0.548	0.99 (0.31–3.17)	0.99	0.88 (0.20–3.90)	0.869
45–64	1.54 (0.94–2.53)	0.085	1.49 (0.75–2.95)	0.329	1.57 (0.45–5.53)	0.483	1.54 (0.29–8.13)	0.612
Education								
No education	Ref.		Ref.		Ref.		Ref.	
Primary	1.89 (1.02–3.47)	0.042	1.87 (0.88–4.00)	0.104	2.22 (0.70–7.01)	0.175	2.20 (0.65–7.41)	0.204
Secondary or more	4.37 (2.56–7.48)	<0.001	3.48 (1.67–7.25)	0.001	2.63 (0.86–8.03)	0.09	2.28 (0.62–8.33)	0.213
Occupation								
Housewife	Ref.		Ref.		Ref.		Ref.	
Garments worker / Housemaid	0.42 (0.24–0.71)	0.001	0.82 (0.43–1.58)	0.559	–		–	
Other work^c^	0.93 (0.62–1.41)	0.748	0.98 (0.62–1.54)	0.93	2.32 (0.77–7.02)	0.135	2.31 (0.71–7.48)	0.162
Age at Marriage (in years)								
≤ 14	Ref.		Ref.		Ref.		Ref.	
15–19	2.09 (1.36–3.21)	0.001	2.18 (1.32–3.60)	0.002	1.14 (0.50–2.63)	0.754	1.11 (0.44–2.78)	0.823
≥ 20	3.02 (1.88–4.84)	<0.001	2.83 (1.61–5.00)	<0.001	1.45 (0.43–4.89)	0.546	1.24 (0.32–4.73)	0.756
Husband’s Education								
No Education	Ref.		Ref.		Ref.		Ref.	
Primary	1.13 (0.55–2.32)	0.743	0.53 (0.22–1.26)	0.149	2.52 (0.80–7.94)	0.114	2.80 (0.84–9.30)	0.092
Secondary or more	2.07 (1.17–3.67)	0.013	0.69 (0.34–1.42)	0.314	2.54 (0.91–7.10)	0.075	2.42 (0.76–7.71)	0.134
Parity								
0	Ref.		Ref.		Ref.		Ref.	
1–2	0.86 (0.45–1.64)	0.654	0.90 (0.44–1.82)	0.768	0.81 (0.17–3.94)	0.794	0.90 (0.18–4.47)	0.899
3 +	0.92 (0.47–1.80)	0.797	1.50 (0.66–3.37)	0.331	1.15 (0.21–6.30)	0.875	1.57 (0.27–9.15)	0.614
Socioeconomic Status^d^								
Low (<10,000)	Ref.		Ref.		Ref.		Ref.	
Medium (10,000–19,999)	1.49 (0.93–2.39)	0.095	1.20 (0.70–2.03)	0.511	0.55 (0.23–1.30)	0.176	0.49 (0.20–1.20)	0.119
High (20,000+)	3.62 (2.25–5.82)	<0.001	2.25 (1.28–3.95)	0.005	0.83 (0.19–3.65)	0.809	0.63 (0.14–2.92)	0.554
Oral Pill Usage								
Never	Ref.		Ref.		Ref.		Ref.	
Ever	0.96 (0.66–1.38)	0.819	1.02 (0.67–1.55)	0.93	0.51 (0.21–1.23)	0.135	0.43 (0.17–1.01)	0.079
Abortion History								
Never	Ref.		Ref.		Ref.		Ref.	
Ever	0.95 (0.70–1.30)	0.823	0.84 (0.57–1.23)	0.363	1.46 (0.48–4.40)	0.503	1.17 (0.36–3.80)	0.799
Condom Usage								
Never	Ref.		Ref.		Ref.		Ref.	
Ever	1.54 (1.10–2.15)	0.012	1.13 (0.79–1.62)	0.515	0.87 (0.39–1.95)	0.741	0.74 (0.31–1.76)	0.498

^a^Adjusted for age

^b^Adjusted for age, education, occupation, age at marriage, parity, oral pill usage, abortion history, socioeconomic status (income), condom use, husband’s education

^c^Includes government and private service, business, daily wager, agriculture, tailor, poultry, handicrafts and tutor

^d^Monthly Expenditure BDT, 1US$ = 78 BDT

## Discussion

In Bangladesh, cervical cancer remains the second most common cancer among women despite global advances in its prevention and treatment. Knowledge of cervical cancer and primary prevention through vaccination is low, indicating a major public health concern for the nation. Our study found that while a large majority of participants (~90%) were aware of cervical cancer, less than 10% had in-depth knowledge of the causes of cervical cancer and potential preventive measures in place. Additionally, participants reported very limited knowledge of the HPV vaccine, particularly rural women. This is the first population-based study conducted in Bangladesh to demonstrate widespread acceptance of HPV vaccination among ever-married adult Bangladeshi women, for both themselves and for their daughters.

To our knowledge, only one other study, the Bangladesh Midlife Women’s Health Study (BMWHS) has been previously conducted in Bangladesh to assess women’s knowledge of cervical cancer [21]. However, the focus of the BMWHS’s research question was on cervical cancer screening and there was no assessment of awareness or acceptability of HPV vaccination presented. Eighty-one percent of the study population of BMWHS reported to have ever heard of cervical cancer, which is lower than both our rural (90%) and urban population’s (93%) awareness. The investigators of this study did not delve further into knowledge of cervical cancer and limited their assessment to five questions which focused on cervical cancer screening. In contrast to our study, the investigators reported that 8.3% of their study population had previously undergone cervical cancer screening. This is particularly interesting as the majority (73%) of their study sample was from rural areas of Bangladesh. In our study, only 3.1% of urban and 0.7% of rural women reported to have ever undergone cervical cancer screening. Findings from our analysis regarding screening rates are comparable to other surveys conducted in developing countries, particularly the South-Asian subcontinent [25].

The results of our cross-sectional study are consistent with other assessments of knowledge and acceptability of HPV vaccine knowledge conducted in low- and middle-income countries. Previous studies have reported both low levels of cervical cancer knowledge and high willingness to receive the HPV vaccine to prevent the development of cervical cancer from HPV [19, 26–31]. For example, in Nepal, previous reports have indicated that overall about half of women were aware of cervical cancer, with a higher awareness among urban Nepali women [19]. In-depth knowledge of cervical cancer was low, however, willingness to have their children vaccinated against HPV was high. In India, levels of knowledge of cervical cancer and acceptance of the HPV vaccine vary vastly by region [32]. In southern India, previous reports have shown that about one-third of women have heard of HPV while only 15% have heard of cervical cancer [33]. Additionally, only 46 % of women were accepting of vaccination. Research conducted in northern Indian among young school-attending girls (12–22 years of age) depicts low awareness (15%) of HPV and cervical cancer, and low acceptance (13%) of the HPV vaccination [34]. Although cervical cancer is the second-leading cause of cancer deaths among women in India [6] and the HPV vaccine is available in the Indian market, uptake is low due to low awareness and knowledge. Efforts should be made in Bangladesh to avoid a similar scenario and resources should be allocated to a wide-spread and culturally sensitive educational campaign to market the HPV vaccine as a vaccine against cancer for maximum uptake.

The results of our study provide important insight into sources of knowledge commonly utilized by Bangladeshi women to obtain information related to health, specifically cervical cancer and the HPV vaccine. The majority of both urban and rural participants reported to have heard of cervical cancer from neighbors, family members, and television programs. Future preventive programming can utilize television as a media source to reach a broader audience [35]. Data from this study also showed the minimal impact health professionals have on the population in terms of cervical cancer education: Only 12% of urban and 8% women reported to have heard of cervical cancer from a physician. Efforts should be made to coordinate all stakeholders, including physicians and other health professionals, in the implementation of successful HPV vaccine programs. Future research should assess levels of knowledge of cervical cancer and its preventative services among health care providers. This is of particular significance as previous literature shows health professionals play a key role in vaccine uptake and that physician recommendation is the strongest predictor of HPV vaccine uptake among adolescents [36].

A limited number of participants were able to identify different risk factors of cervical cancer. Participants who did provide a response for this question most frequently chose unhygienic menstrual practices, sexual intercourse, and a sexual relationship with someone other than their husband as potential ways to develop cervical cancer. Low condom use was not often chosen, indicating that the population is not aware of the etiology of HPV and cervical cancer. This may limit future HPV vaccine programming, as the population may not recognize the association between sexually transmitted infections (STIs) and cervical cancer, and opt out of receiving the vaccination. However, previous literature has also indicated that the association between STIs and cervical cancer has hindered efforts to promote the HPV vaccine in the population [37]. In several countries, a frequently identified barrier to HPV vaccination has been parental fears that the vaccine may lead to sexual promiscuity [38]. This is a vital concern and practitioners involved in primary care, school-based health services, and adolescent health should be engaged to avoid the potential spread of misinformation regarding the safety and efficacy of the vaccine. Future implementation efforts should include culturally appropriate educational interventions through the media, particularly the television, in order to better inform the population of the association between HPV and cervical cancer.

Similarly, only a small proportion of participants were able to provide responses regarding preventive methods against cervical cancer. The majority of participants chose medications, one-time examination by doctor, cleanliness, and maintaining a monogamous sexual relationship with their husband. Only a limited number of the respondents were able to correctly choose vaccination and regular examination by a physician. This finding further underscores the need for widespread educational interventions on cervical cancer and associated factors, such as risk behaviors and preventive methods. Such educational interventions can be targeted to groups who were identified to be less likely to have heard of cervical cancer based on multivariable analyses. These groups include: younger women in urban and rural areas, women with either no or low education in urban areas and rural areas, urban women with low income, and urban women who do not use condoms.

Although findings from this study are novel and timely for successful HPV vaccine program implementation, several limitations should be taken into consideration when interpreting these results. Participants included in this study resided in surveillance sites of icddr,b. where they have frequently participated in various public health initiatives and research projects. This frequent exposure to public health programming may explain the population’s widespread willingness to receive the HPV vaccination or a vaccine to prevent cervical cancer. As such, this sample may not be representative of the entire population, however, one can expect even lower knowledge in the broader population outside of the surveillance site. Additionally, questions included in this survey were close-ended; open-ended questions may have provided the opportunity to better assess cervical cancer and HPV related knowledge, perceived risk and awareness, especially when no prior data were available for this population.

The high level of willingness to receive a vaccine to prevent cervical cancer despite low knowledge of cervical cancer presents potential ethical issues and underscores the need to provide educational programs. This will ensure that Bangladeshi women are able to make informed decisions on their health and that of their families. Additionally, in the absence of accessible regular screening programs, the implementation of an HPV immunization program should be a public health priority in Bangladesh. In our study population, only 1.9% of the entire sample had previously undergone screening for cervical cancer. This finding is consistent with previous literature published in 2012, which estimated that only 1.1% of the women in Bangladesh had previously undergone cervical cancer screening based on data collected in 2003 [25]. Based on these results, it is reasonable to conclude that rates of cervical cancer screening have not improved over the last decade in Bangladesh. Previous studies have shown that low awareness and socioeconomic barriers can potentially lead to underutilization of screening services for women. Education of women by healthcare workers is an important factor for increasing screening rates [39]. Future research is needed to explore the level of knowledge of cervical cancer in other populations at high risk.

## Conclusions

This study provides the first population-based assessment of willingness to receive the HPV vaccine. These findings are timely as the Government of Bangladesh has recently obtained support from GAVI to provide a national HPV vaccine program to school-aged adolescent girls. The findings from this study provide the necessary country-specific evidence for the development of this program. Despite high levels of awareness of cervical cancer, in-depth knowledge of causes of cervical cancer and how it can be prevented is low. Additionally, cervical cancer screening was very low in this population. These findings underscore the necessity for culturally appropriate and targeted educational interventions to improve knowledge of cervical cancer causes and its primary prevention through the HPV vaccine. Despite low knowledge about the disease and its prevention, there was a high level of willingness to receive a vaccine to prevent cervical cancer. As such, this survey suggests that the HPV vaccine would likely be an accepted addition to routine vaccinations in Bangladesh. Findings from this study have important implications for designing and implementing HPV vaccine programs, and educational efforts in the country.

## Abbreviations

aOR: Adjusted Odds Ratio
BDT: Bangladeshi Taka
BMWHS: Bangladesh Midlife Women’s Health Study
CI: Confidence Interval
GAVI: Global Alliance for Vaccines and Immunizations
HPV: Human Papillomavirus
icddr,b: International Center for Diarrheal Disease Research, Bangladesh
OR: Odds Ratio
SES: Socioeconomic status
STIs: Sexually transmitted infections
VIF: Variance inflation factor
